# Incidental Diagnosis of Adult Beta-Thalassemia With Point-of-Care Ultrasound in the Emergency Department: A Case Report

**DOI:** 10.7759/cureus.12063

**Published:** 2020-12-13

**Authors:** Jasmine L Dennis, Dustin Morrow, Julia A Cupp

**Affiliations:** 1 Emergency Medicine, University of South Carolina School of Medicine at Greenville, Greenville, USA; 2 Internal Medicine, University of South Carolina School of Medicine at Greenville, Greenville, USA

**Keywords:** thalassemia, beta-thalassemia, ultrasound, ultrasonography, pocus, splenomegaly, emergency

## Abstract

Beta-thalassemia is an autosomal recessive hemoglobinopathy that can result in microcytic hypochromic anemia, splenomegaly, hypercoagulability, and long-term sequelae. Beta-thalassemia intermedia, specifically, is diagnosed based on the moderate severity of illness, which does not carry the early symptomatic urgency of beta-thalassemia major, although patients of both often become chronically or intermittently transfusion-dependent. A presenting symptom may be splenomegaly, which is most efficiently detected with a combination of physical examination and point-of-care ultrasound (POCUS).

We present the case of a 25-year-old male patient with no significant past medical history who presented to the emergency department with abdominal discomfort for one week. The history of present illness, review of systems, and physical exam were nonrevealing. An ultrasound was performed to rule out renal colic; however, he was incidentally found to have an enlarged and infarcted spleen. This unexpected discovery warranted a laboratory workup, which indicated beta-thalassemia intermedia. His diagnosis was confirmed with serum protein electrophoresis and he was thereafter followed by hematology.

Beta-thalassemia intermedia can present suddenly in adulthood, despite a benign past medical history. Splenomegaly may be a presenting symptom and can be effectively detected with a physical exam plus POCUS. Failure to detect these subtleties can lead to potentially life-threatening conditions such as profound anemia, thromboembolic accidents, pulmonary hypertension, and pathological fractures. This case demonstrates the importance of utilizing POCUS in combination with a physical examination to attain a comprehensive perspective of anatomy, even in those patients fast-tracked in the emergency department.

## Introduction

Beta-thalassemia is an autosomal recessive hemoglobinopathy caused by a mutation in one or both beta-globin loci in erythrocytes [1]. This mutation can result in microcytic hypochromic anemia, hypercoagulability, splenomegaly, extramedullary hematopoiesis, gallstones, and pathological fractures; most untreated patients die from high-output heart failure [1].

Beta-thalassemia intermedia is diagnosed by the severity of illness, which does not carry the symptomatic urgency of beta-thalassemia major, although both can become chronically or intermittently transfusion-dependent [2]. Patients with beta-thalassemia intermedia often have a hemoglobin level ranging between 7-10 g/dL and become symptomatic during periods of systemic stress such as pregnancy, infection, or other illnesses [2].

Splenomegaly is a relatively early symptom of beta-thalassemia intermedia that is possibly detected by physical exam. However, physical examination of splenomegaly is more specific than sensitive and is best used to rule in a diagnosis already suspected [3]. Since findings may be limited by body habitus, general anatomy, and physician experience, many physicians turn by default to imaging modalities such as ultrasonography, CT, or nuclear liver-spleen imaging [4].

## Case presentation

We present the case of a 25-year-old male with no reported significant past medical history who presented to the emergency department, per the insistence of his girlfriend, with left-sided upper abdominal and back pain for one week. The pain, described as an “air bubble," was 6/10 on the Numeric Rating Score for pain at its worst, alleviated by drinking water, and exacerbated by deep breathing. He initially attributed the discomfort to flatus from fried chicken and reported that simethicone had not improved the discomfort. He denied fever, weight loss, night sweats, nausea, vomiting, diarrhea, or dysuria. He also denied recent illness or travel.

The patient-reported family history was notable for a brother with possible sickle cell trait and a mother with anemia of unknown etiology. This prompted further probing of the patient’s medical history, during which he did endorse having been told he had sickle cell trait, although he had never been tested or received a transfusion.

**Figure 1:**
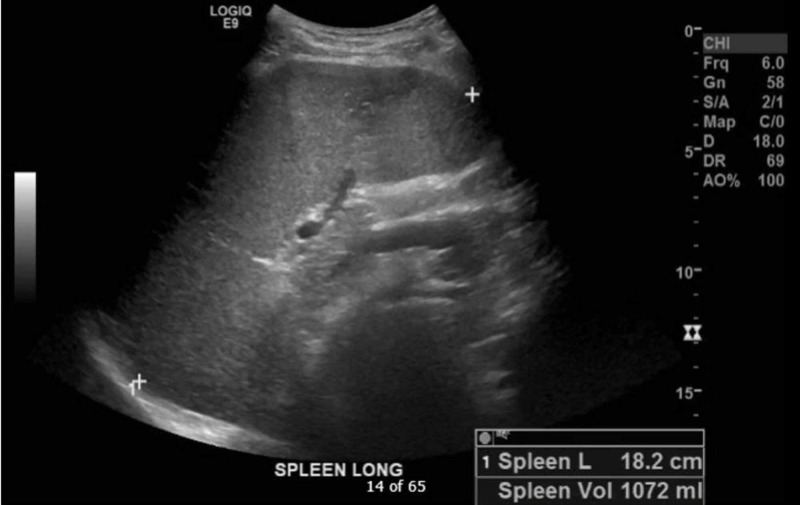
Splenomegaly indicated by spleen length at 18.2 cm with a splenic volume of 1,072 mL

**Figure 2:**
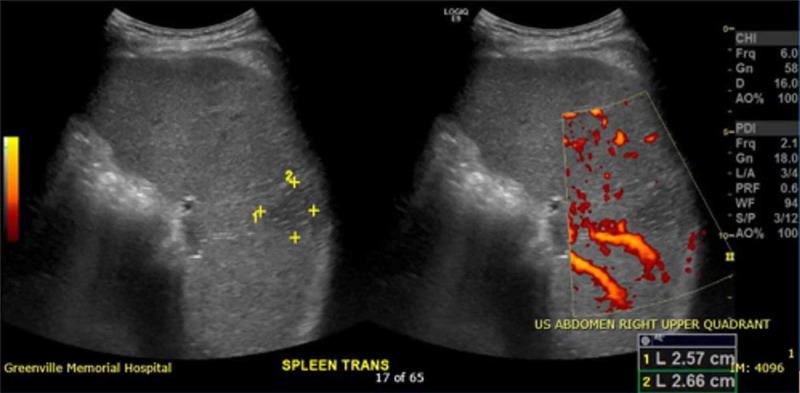
Splenic wedge-shaped hypoechogenic lesion, 2.57 x 2.66 cm, indicative of absent blood flow in the periphery of the spleen due to infarction

The general impression of this man was a healthy-appearing young adult in mild to moderate abdominal discomfort with no worrisome signs, symptoms, or past medical history. The patient expressed minimal concern for his symptoms; however, his girlfriend insisted that the abdominal pain was abnormal for him. Besides, point-of-care ultrasound (POCUS) was utilized to exclude the presence of free fluid in the abdomen that might suggest a more serious cause of abdominal pain in the left upper quadrant. Unexpectedly, and beyond the scope of limited POCUS, splenomegaly (Figure 1) with areas of hypoechogenicity indicative of splenic infarcts (Figure 2) was appreciated by the emergency provider, which prompted further investigation.

This unexpected discovery suggested a pathologic etiology of his abdominal pain and enabled the emergency physician to convince the patient to stay for further testing. Bloodwork revealed microcytic anemia, thrombocytopenia, hyperbilirubinemia, and a high reticulocyte count consistent with hemolysis. HIV antibody, heterophile antibody test, and fecal occult blood tests were negative. Laboratory tests pending at the time of discharge, which were also found to be negative, included paroxysmal nocturnal hemoglobinuria, factor V Leiden mutation, and prothrombin 20210 mutation.

**Figure 3:**
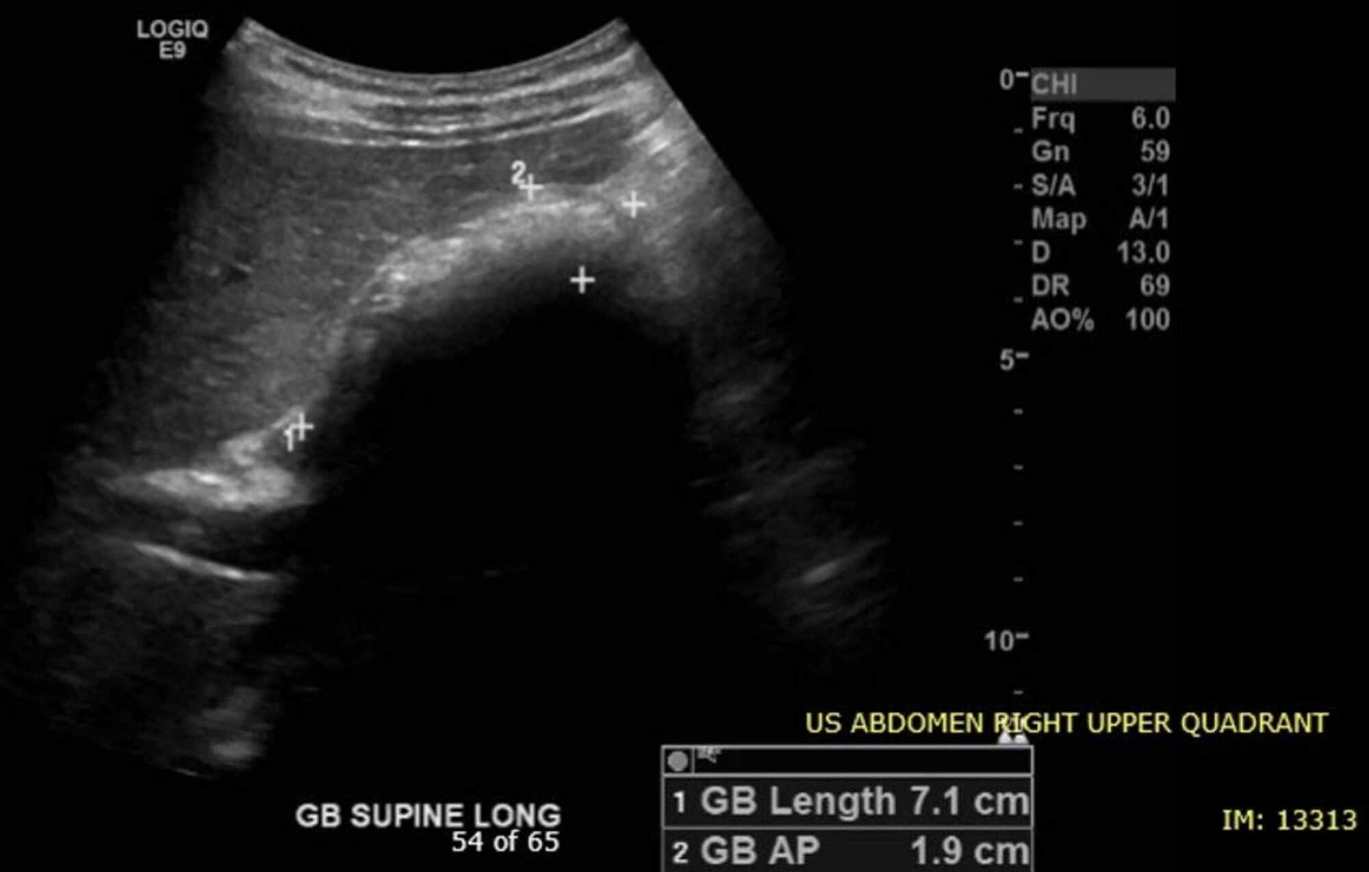
Gallbladder with multiple gallstones Gallbladder length is within normal limits at 7.1 cm

A comprehensive radiology study was ordered, which confirmed the POCUS findings of pronounced splenomegaly with multifocal splenic infarcts, as well as uncomplicated cholelithiasis (Figure 3).

Due to his low hemoglobin of 6.9 g/dL, the patient was given one unit of packed red blood cells with an appropriate increase in his subsequent hemoglobin to 8.2 g/dL. Hematology was consulted, and a hypercoagulability and hemoglobinopathy workup was performed. The patient was then discharged with prescriptions for cyanocobalamin tablets, folate tablets, and diphenhydramine for itching after his transfusion. He was advised to follow up with outpatient hematology for pending laboratory results, continued management of his presumed newfound thalassemia, and referral for iron chelation therapy.

## Discussion

The serum laboratory evaluation of this patient was interpreted as profoundly microcytic anemia with target cells, teardrop cells, and high reticulocyte count indicative of functional bone marrow compensation for extravascular hemolysis. Additionally, the decreased hemoglobin A1 (normal adult hemoglobin made of alpha- and beta-globin chains) with profoundly increased hemoglobin F (fetal hemoglobin made of alpha-and gamma-globin chains) and increased hemoglobin A2 (alternative adult hemoglobin made of alpha- and delta-globin chains) suggested a deficiency of beta-globin synthesis, further supporting a diagnosis of beta-thalassemia.

Beta-thalassemia intermedia, also known as non-transfusion-dependent thalassemia, was coined to describe clinical manifestations that are not severe enough to be classified as “major” yet often still require transfusions [2]. It is important to detect patients with beta-thalassemia intermedia as it is known to carry the comorbidity of hypercoagulability, with paradoxically increased predisposition for thrombosis compared to beta-thalassemia major, with risks including portal vein thrombosis, pulmonary embolism, and stroke [5]. As an autosomal recessive disease, detection not only improves the health of the patient but also prompts family members to be screened and receive an appropriate follow-up, if needed. Common symptoms of beta-thalassemia intermedia are thrombophilia, extramedullary hematopoiesis, cholelithiasis, and splenomegaly [6], all of which were experienced by this patient.

One of the most important aspects of this case was abdominal pain, which was likely attributed to splenic infarcts from the patient's hypercoagulable state, and splenomegaly from hemolysis and extramedullary hematopoiesis. Traditionally, physicians have been taught to screen for anemia or splenomegaly, both potentially associated with abdominal pain, by physical examination. However, these examinations alone do not lend themselves to be reliable screening tools. Pertaining to anemia physical examination findings, it has been concluded that the absence of pallor does not rule out anemia and is, therefore, not useful in ruling out suspicious diagnoses. In a study by Nardone et al., pallor at the conjunctiva, face, or palms yielded unconvincing sensitivities ranging from 0.53-0.65. The pallor of all three anatomic sites together yielded a specificity of 0.95, which could be used to confirm anemia, albeit not as a consistently useful diagnostic tool [7]. Pertaining to splenomegaly physical examination findings, results are often limited by body habitus, physician experience, or anatomical variations. Physical examination of splenomegaly is more specific than sensitive and is best used to rule in a diagnosis. For greater certainty in detecting splenomegaly on physical examination, Simel et al. have suggested using Traube's space percussion, followed by supine one-handed palpation, which may be superior to percussion in lean patients [4]. For the most reliable detection of splenomegaly, a combination of physical examination and POCUS demonstrated a strong sensitivity of 100%, while physical examination alone was determined to have a sensitivity of only 40% [3]. Thus, it can be concluded that a patient suspected of suffering from splenomegaly should receive percussion, palpation, and POCUS of the abdominal left upper quadrant. Based on our patient's height and male sex, the predicted upper limit of normal spleen volume was 300 mL, and the predicted upper limit of normal spleen length was 13.1 cm [8]. This patient’s spleen volume was measured at 1,072 mL and spleen length measured at 18.2 cm, both markedly beyond the upper limit of normal.

## Conclusions

Abdominal pain poses a frequent challenge to physicians, as the severity and clinical consequences of potential etiologies are vast. Amidst these frequent and variable complaints, splenomegaly is an important diagnosis to make, although it can be subtle and difficult to detect with history and physical exam alone. Splenomegaly is indicative of a broad range of potentially clinically significant underlying pathology such as beta-thalassemia intermedia as seen in our patient. Beta-thalassemia intermedia is an example of a rare disease that may present in healthy older children or adults and can have fatal consequences if not detected, such as profound anemia, thromboembolic accidents, pulmonary hypertension, and pathological fractures. This case demonstrates the importance of utilizing both physical examination and POCUS together in patients suspected of splenic pathology in the emergency department.
